# First-principles study on the electrical and thermal properties of the semiconducting Sc_3_(CN)F_2_ MXene†

**DOI:** 10.1039/c8ra03424a

**Published:** 2018-06-19

**Authors:** Kan Luo, Xian-Hu Zha, Yuhong Zhou, Zhansheng Guo, Cheng-Te Lin, Qing Huang, Shenghu Zhou, Ruifeng Zhang, Shiyu Du

**Affiliations:** School of Chemical Engineering, East China University of Science and Technology Shanghai China zhoushenghu@ecust.edu.cn; Engineering Laboratory of Specialty Fibers and Nuclear Energy Materials, Ningbo Institute of Materials Technology and Engineering, Chinese Academy of Sciences Ningbo Zhejiang China dushiyu@nimte.ac.cn; Shanghai Institute of Applied Mathematics and Mechanics, Shanghai University Shanghai China; Key Laboratory of Marine Materials and Related Technologies, Zhejiang Key Laboratory of Marine Materials and Protective Technologies, Ningbo Institute of Materials Technology and Engineering, Chinese Academy of Sciences Ningbo Zhejiang China; School of Materials Science and Engineering, Beihang University Beijing China

## Abstract

The two-dimensional materials MXenes have recently attracted interest for their excellent performance from diverse perspectives indicated by experiments and theoretical calculations. For the application of MXenes in electronic devices, the exploration of semiconducting MXenes arouses particular interest. In this work, despite the metallic properties of Sc_3_C_2_F_2_ and Sc_3_N_2_F_2_, we find that Sc_3_(CN)F_2_ is a semiconductor with an indirect band gap of 1.18 eV, which is an expansion of the semiconducting family members of MXene. Using first-principles calculations, the electrical and thermal properties of the semiconducting Sc_3_(CN)F_2_ MXene are studied. The electron mobilities are determined to possess strong anisotropy, while the hole mobilities show isotropy, *i.e.* 1.348 × 10^3^ cm^2^ V^−1^ s^−1^ along *x*, 0.319 × 10^3^ cm^2^ V^−1^ s^−1^ along the *y* directions for electron mobilities, and 0.517 × 10^3^ cm^2^ V^−1^ s^−1^ along *x*, 0.540 × 10^3^ cm^2^ V^−1^ s^−1^ along the *y* directions for hole mobilities. The room-temperature thermal conductivity along the *Γ* → *M* direction is determined to be 123–283 W m^−1^ K^−1^ with a flake length of 1–100 μm. Besides, Sc_3_(CN)F_2_ presents a relatively high specific heat of 547 J kg^−1^ K^−1^ and a low thermal expansion coefficient of 8.703 × 10^−6^ K^−1^. Our findings suggest that the Sc_3_(CN)F_2_ MXene might be a candidate material in the design and application of 2D nanoelectronic devices.

## Abstract 2

The two-dimensional semiconducting Sc_3_(CN)F_2_ MXene presents relatively high carrier mobilities, specific heat and low thermal expansion coefficient from DFT calculations, and produces a good application prospect for nanoelectronic devices.

## Graphical abstract

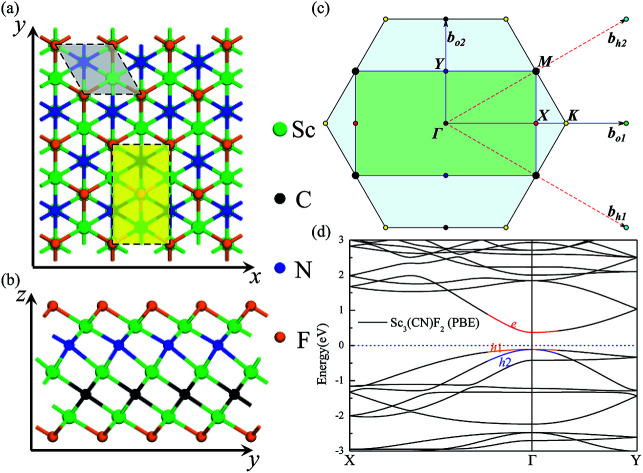

## Introduction

MXenes, a new class of two dimensional transition metal carbides or nitrides with the chemical formula of M_*n*+1_X_*n*_ (M = Sc, Ti, V, Cr, Zr, Nb, Mo, Hf, Ta; X = C, N; *n* = 1–3), have been synthesized from the exfoliation process for ternary layered metallic ceramics such as the MAX phases and immediately attracted extensive attention in recent years.^1–5^ The MAX phases are a family of layered compounds with a chemical formula of M_*n*_AX_*n*+1_ (*n* = 1–3), where A includes Al, Si, P, S, Ga, Ge, As, In, and Sn.^6,7^ There are currently over 70 MAX phases known and this family is still growing due to their large number of solid solutions.^8,9^ The terminations on MXenes are typically functionalized by –H, –F, =O, and –OH groups coming from HF or H_2_O.^10–12^ Naguib *et al.*^1^ have denoted the functionalized MXenes as M_*n*+1_X_*n*_T_*x*_, with T standing for the surface-terminating group. Recently, about 20 different MXenes have been reported,^13^ and the family of MXenes has been expanded to double transition metals carbides M′_2_M′′C_2_ and M′_2_M′′_2_C_3_.^14^ The large number of theoretically possible members of the MXene family, the diversity of physical properties among MXenes, and their relative convenience in synthesis merit the attraction of these compounds for novel production methods and 2D material-related potential applications.^15^ For example, Xu *et al.* reported the growth of high quality crystalline MXenes achieved by a chemical vapor deposition technique.^16^ Azofra *et al.* investigated the N_2_ capture and ammonia conversion behaviour of d^2^–d^4^ MXenes,^17^ and the CO_2_ capture and conversion may be another possible application of the MXene materials.^18^ Some of the MXenes are demonstrated to be topological insulators,^19,20^ exhibiting multiple Dirac cones and giant spin–orbit splitting.^21^ Ashton *et al.* compared the thermodynamic stability of 54 MXenes,^22^ finding Sc-based MXenes to be highly stable with F termination, and the low diffusion barriers for Li on fluorinated MXene surfaces^23^ can make Sc_*n*+1_X_*n*_F_2_ MXenes possible candidates for electrode materials in Li-ion batteries. Many of the recent studies on MXenes have been focused on the electronic, magnetic, catalytic or thermoelectric properties.^24–31^ Liu *et al.* systematically explored the electronic properties of Sc-based MXenes by first-principles calculations.^32^ Wang *et al.* investigated the band gap tuning of Sc_2_C MXene for optoelectronic devices by changing the types of surface chemical groups,^33^ and heterostructures based on three different functionalized Sc_2_C MXenes were built to investigate the possible application for nanodevices.^34^ The data from these works suggest that MXenes are promising as electronic devices, for which the semiconducting members are generally desired. However, most MXenes are metallic due to the inheritance of the conducting feature of the electronic band structures in transition metal carbides or nitrides. Therefore, further investigation of these materials is needed, such as the effect of compositional modification on electronic properties as well as structural stability, in order to expand the MXene family, especially for intrinsically semiconducting ones.^35^

In this work, the band structures of three fluorine-functionalized scandium MXenes Sc_3_C_2_F_2_, Sc_3_N_2_F_2_ and Sc_3_(CN)F_2_ are studied using density functional theory (DFT). Here, we demonstrate that Sc_3_(CN)F_2_ is a semiconductor with an indirect band gap of 1.18 eV from the Heyd–Scuseria–Ernzerhof (HSE06) correction. This demonstrates that the design of new semiconducting MXenes is possible. The electronic, carrier mobility and thermal properties of the Sc_3_(CN)F_2_ MXene are also predicted *via* theoretical calculations. The strong anisotropy in electron mobility has been determined. In addition, the relatively high specific heat and low thermal expansion coefficient make Sc_3_(CN)F_2_ a good candidate material for nanoelectronic devices.

## Computational details

The first-principles calculations are carried out based on projector augmented-wave (PAW) potentials^36^ in reciprocal space represented by a generalized gradient approximation (GGA)^37^ in density functional theory with Perdew–Burke–Ernzerhof (PBE) for the exchange–correlation function as implemented in the VASP codes.^38^ Plane-waves with energies up to 550 eV are employed to describe the electronic wave functions, in which the Sc 3p^6^3d^1^4 s^2^, C 2s^2^2p^2^, N 2s^2^2p^3^ and F 2s^2^2p^5^ electrons are considered as valence states. To avoid any artificial interaction between the layers and their images, a 30 Å lattice parameter in the *c*-axis perpendicular to the MXene surface is set. In the optimized structures, the maximum force on each atom is less than 10^−4^ eV Å^−1^. The total energies are converged within 10^−6^ eV. For the structural optimization, the Brillouin zone (BZ) is sampled using a set of *Γ*-centered 12 × 12 × 1 *k*-points. Due to the underestimation of energy band gaps through GGA-PBE,^39^ the non-local HSE06 hybrid functional is also adopted to correct the band gap values.^32,40^

**Fig. 1:**
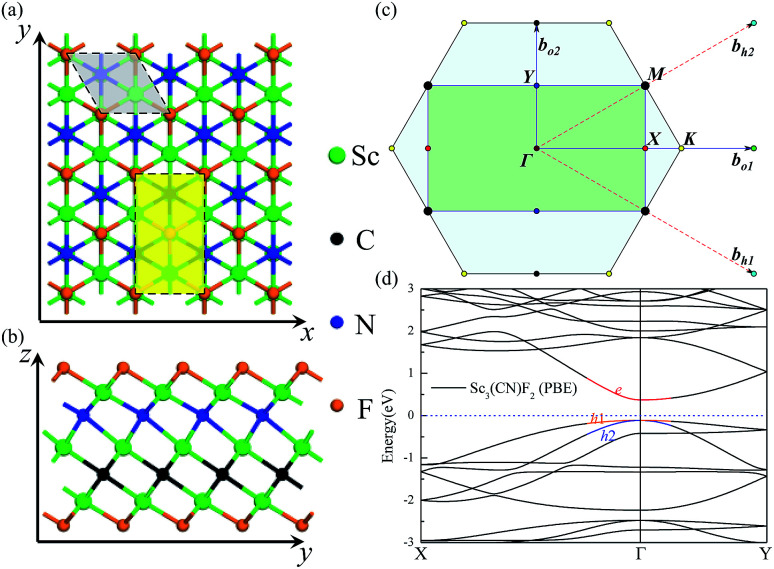
The top view (a) and side view (b) of the Sc_3_(CN)F_2_ MXene. (c) The Brillouin zone of the 2D hexagonal and orthorhombic lattice, the high symmetry routes *Γ* → *K* (*Γ* → *X*) and *Γ* → *M* (*Γ* → *Y*) correspond to the real-space *x* and *y* directions, respectively. (d) The band structure of the Sc_3_(CN)F_2_ MXene based on the orthorhombic cell with the Fermi level located at zero. The atoms are represented by spheres: Sc (green), C (black), N (blue) and F (orange).

The carrier mobilities of the Sc_3_(CN)F_2_ MXene are calculated using the deformation potential (DP) theory^41–43^ based on an orthorhombic unit cell, as the yellow rectangle highlights in Fig. 1(a). The carrier mobility has been calculated according to eqn (1)^44,45^1
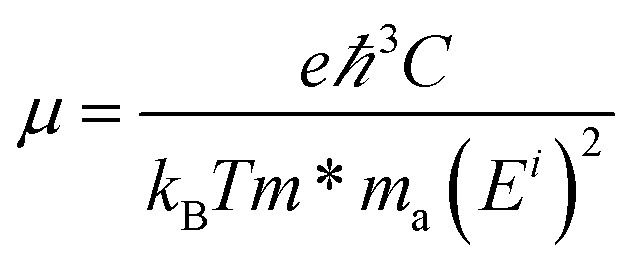
where *ħ* and *k*_B_ are the reduced Planck and Boltzmann constants, respectively. *T* denotes temperature, and m* is the carrier effective mass along the transport direction; *m*_a_ is calculated by 
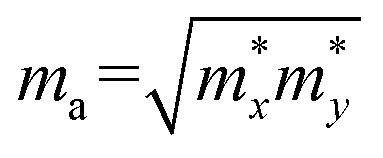
, where 
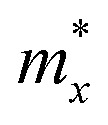
 and 
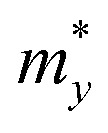
 are the carrier effective masses along the *x* and *y* directions, respectively, as shown in Fig. 1(c). *C* is the elastic modulus along the transport direction, determined by extrapolation based on the relationship of *C*(Δ*a*/*a*)^2^/2 = (*E* − *E*_0_)/*S*_0_, where (*E* − *E*_0_) is the change of the total energy under a small lattice variation Δ*a* from the equilibrium lattice constant *a*_0_ along the transport direction, with a small step size (Δ*a*/*a*_0_ ∼ 0.5%), and *S*_0_ is the area of the lattice in the *xy* plane. Finally, *E*^*i*^ is the deformation potential constant of the valence band maximum (VBM) for holes or the conduction band minimum (CBM) for electrons along the transport direction, calculated by *E*^*i*^ = Δ*V*_*i*_/(Δ*a*/*a*_0_) with Δ*V*_*i*_ as the energy change of the *i*^th^ energy band. The deformation potential constant is estimated as the slope of the linear fitting function between Δ*V*_*i*_ and Δ*a*/*a*_0_.

The thermal conductivities have been calculated from the phonon dispersion of a hexagonal unit cell, as the gray rhombus marks in Fig. 1(a). The phonon thermal conductivity was calculated within the framework of Klemens’ theory^46,47^2
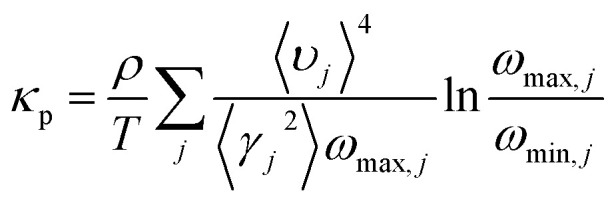
where *ρ* is the mass density, calculated by 
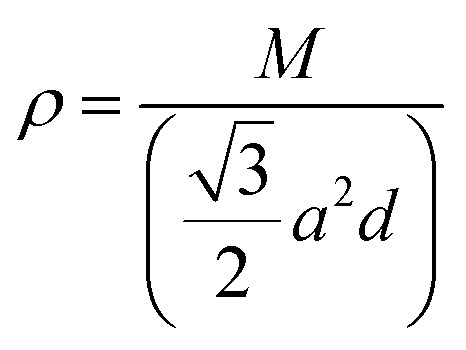
, with *M* being the mass of the MXene unit cell, *a* is the lattice parameter in the *xy* plane, and *d* denoting the MXene layer thickness.^48^ A bilayer Sc_3_(CN)F_2_ MXene structure model is optimized to calculate the layer thickness. The value of *d* = 10.284 Å is measured as the distance between two middle layer Sc atom planes in the bilayer Sc_3_(CN)F_2_ MXene. To accurately describe the interlayer interaction of the bilayers for Sc_3_(CN)F_2_, a zero damping van der Waals (vdW) correction (DFT-D3) of Grimme^49^ has been adopted. *υ*_*j*_, *ω*_max,*j*_ and *ω*_min,*j*_ are the group velocity and the maximum and minimum circular frequency of each *j*^th^ branch, respectively. Due to the finite flake length *L*, the term of *ω*_min,*j*_ is redefined as 
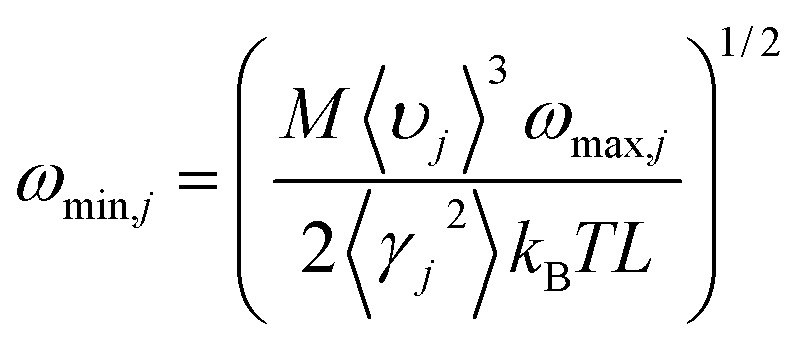
, where *γ*_*j*_ is the average value of the branch Grüneisen parameter, and 〈*γ*^2^_*j*_〉 in eqn (2) is estimated by 
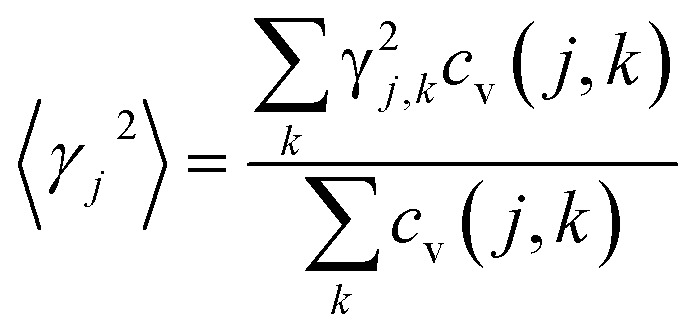
. Phonopy software^50^ combined with the VASP code is utilized for phonon dispersion calculations. The theoretical calculation is performed with density functional perturbation theory (DFPT),^51^ and a 6 × 6 × 1 *k*-points mesh based on a 2 × 2 × 1 super-cell is adopted for calculating the dynamical matrix. The thermal expansion coefficient *α* is investigated based on the Grüneisen approximation,^52^
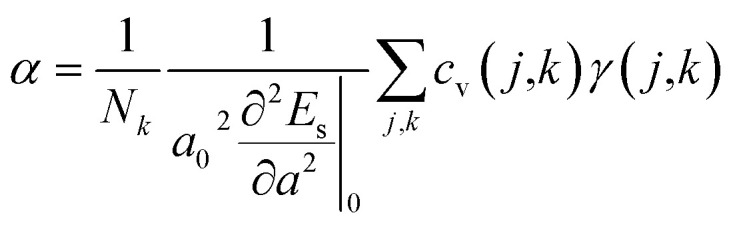
. Here, *N*_*k*_ is the *k*-point number adopted in plotting the phonon spectrum, which is equal to 120 in our calculations; *E*_s_ is the strain energy; *c*_v_(*j,k*) is the (*j,k*) mode contribution to the heat capacity, 
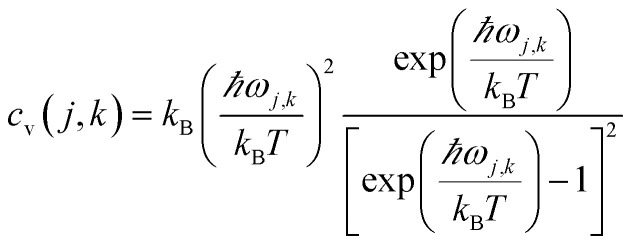
. The Specific heat *c* is proportional to the heat capacity as 
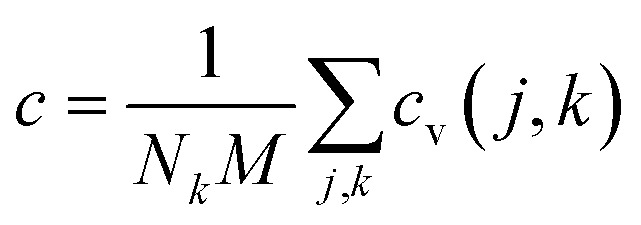
.^53^

The computational parameters and methods applied in calculating the carrier mobility and thermal properties have been tested in our previous works on Sc_2_CF_2_, Sc_2_C(OH)_2_ (ref. 54) and Hf_2_CO_2_ (ref. 55) MXenes. The predicted thermal conductivity of graphene in our previous calculation (4.76 × 10^3^ W m^−1^ K^−1^, based on a 5 μm flake length at room temperature) is consistent with the experimental results.^56^

## Results and discussion

**Fig. 2:**
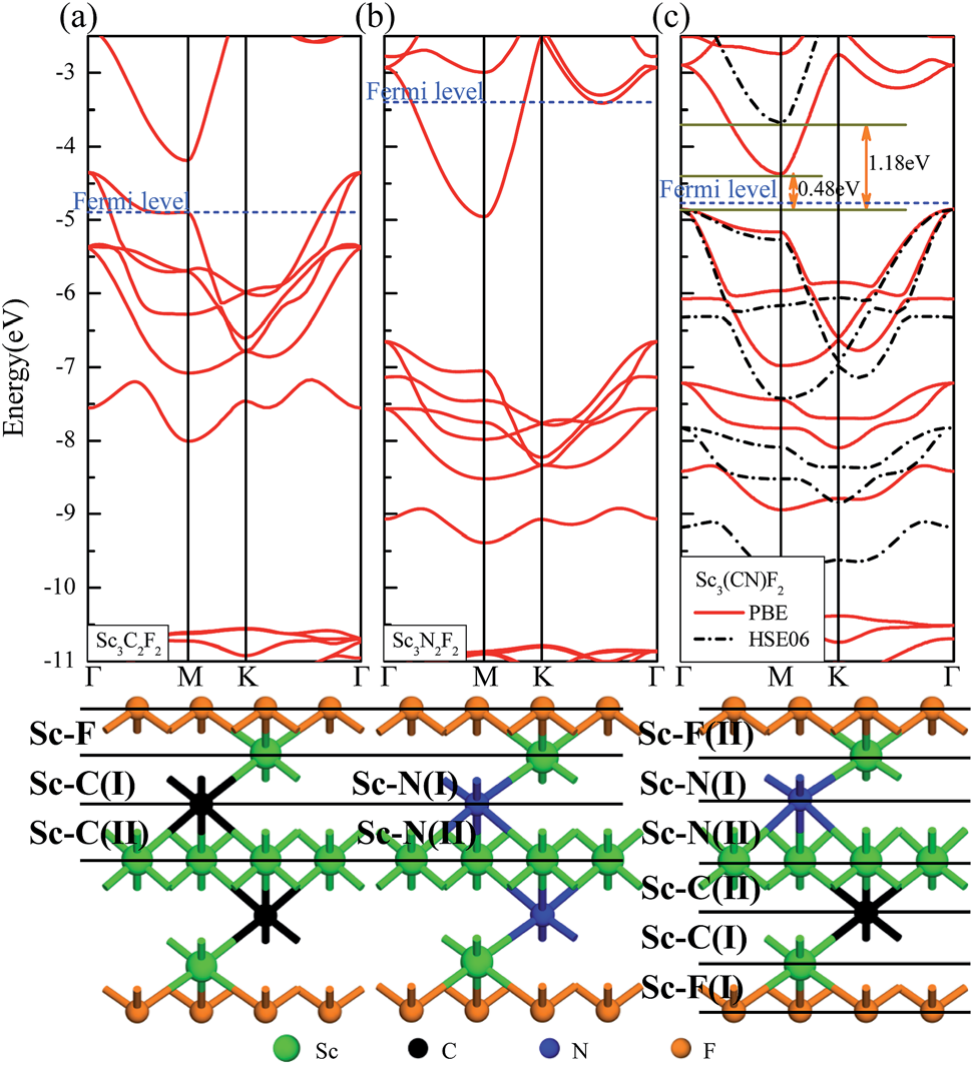
Band structures of three F terminated MXenes Sc_3_C_2_F_2_ (a), Sc_3_N_2_F_2_ (b) and Sc_3_(CN)F_2_ (c), and the vacuum energy is set as zero. Red solid and black dotted lines represent electronic energy bands from GGA-PBE and HSE06 respectively. The side view of the Sc_3_C_2_F_2_, Sc_3_N_2_F_2_ and Sc_3_(CN)F_2_ MXenes are shown below each band structure figure respectively.

**Fig. 3:**
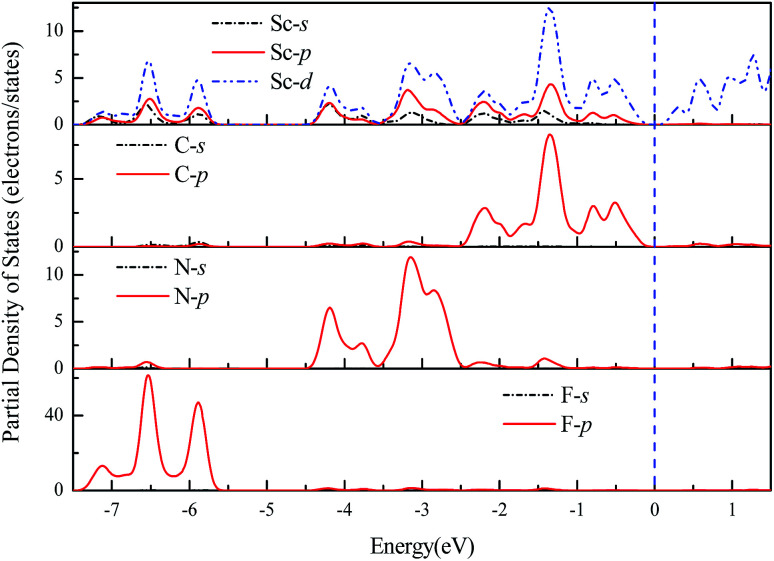
Partial density of states for the Sc_3_(CN)F_2_ MXene.

The geometries and band structure properties of the F terminated MXenes Sc_3_C_2_F_2_, Sc_3_N_2_F_2_ and Sc_3_(CN)F_2_ are investigated using DFT calculations. As 2D hexagonal materials, the MXenes possess two high-symmetry routes, namely, the *y* and *x* directions.^57^ The top view and side view of the Sc_3_(CN)F_2_ MXene are shown in Fig. 1(a) and (b). The Sc_3_C_2_F_2_ and Sc_3_N_2_F_2_ have similar structures to Sc_3_(CN)F_2_ and their side view diagrams are also shown in Fig. 2(a) and (b), respectively. According to our structure models, the *x*-axis coincides with the *x* direction, and the *y*-axis lies along the *y* direction. The *Γ* → *K* (*Γ* → *X*) and *Γ* → *M* (*Γ* → *Y*) vectors in the Brillouin zone correspond to the real-space *x* and *y* directions as shown in Fig. 1(c), respectively. The two carbon or nitrogen layers are sandwiched between three Sc layers, and two fluorine layers are projected onto the central Sc layer. Table 1 lists the lattice constants, formation energies and atomic layer distances marked in Fig. 2. The optimized lattice constant of Sc_3_(CN)F_2_ is similar to that of Sc_3_C_2_F_2_, and the formation energy is between that of Sc_3_C_2_F_2_ and Sc_3_N_2_F_2_. As with the result of the substituted C/N atoms, the Sc–F atomic layer distances are only slightly affected, while the Sc–C and Sc–N distances show notable variations, especially for the bonds with center Sc atoms (labelled as II in Table 1). The band structures of Sc_3_C_2_F_2_, Sc_3_N_2_F_2_ and Sc_3_(CN)F_2_ are also provided in Fig. 2 (vacuum energy is set as zero). Both Sc_3_C_2_F_2_ and Sc_3_N_2_F_2_ exhibit metallic properties with the Fermi level crossed by energy bands and with band gaps above/below the Fermi levels, while Sc_3_(CN)F_2_ is determined to be a semiconductor with an indirect band gap of 1.18 eV from HSE06. From the band structure plots, the three F terminated MXenes also exhibit similar shapes near the Fermi level despite the difference in band gaps. The band gap can also be observed from the partial density of states (PDOS) plot for Sc_3_(CN)F_2_ in Fig. 3. From the figure, Sc and N overlap from −4.5 to −2.5 eV, while Sc and C are from −2.5 to 0 eV (forming CBM) near the Fermi level. Sc_3_C_2_F_2_ and Sc_3_(CN)F_2_ show similar Fermi level energy; since the Sc–C bonds are strengthened in Sc_3_(CN)F_2_ as seen from the reduction of Sc–C bond lengths, the N atoms substitutions lowers the energy of Sc–C hybrid bands forming VBM bands in Sc_3_(CN)F_2_ relative to that in Sc_3_C_2_F_2_ around the Fermi level. Similarly, the CBM energy of Sc_3_(CN)F_2_ is raised relative to the corresponding bands in Sc_3_N_2_F_2_. These result in a rise of the band gap for Sc_3_(CN)F_2_ at a particular C/N ratio. This implies band engineering can be achieved in Sc-based MXenes by the structural design of the MXene, thus expanding the group of semiconducting MXenes.

**Table 1:** The lattice constants, formation energies and atomic layer distances obtained by structure relaxation

MXene	*a* (Å)	Formation energy (eV per atom) (competing phase)	Atomic layer distance (Å)					
			Sc–F		Sc–C		Sc–N	
Sc_3_C_2_F_2_	3.243	−1.77 (0.063; ScF_3_, Sc_4_C_3_, C)	1.137	1.282(I)	1.413(II)	—	—	
Sc_3_N_2_F_2_	3.190	−2.70 (−0.057; ScF_3_, ScN, Sc)	1.175	—	—	1.145(I)	1.354(II)	
Sc_3_(CN)F_2_	3.244	−2.29 (−0.052; ScF_3_, Sc_4_C_3_, ScN)	1.133(I)	1.155(II)	1.273(I)	1.264(II)	1.066(I)	1.524(II)

**Fig. 4:**
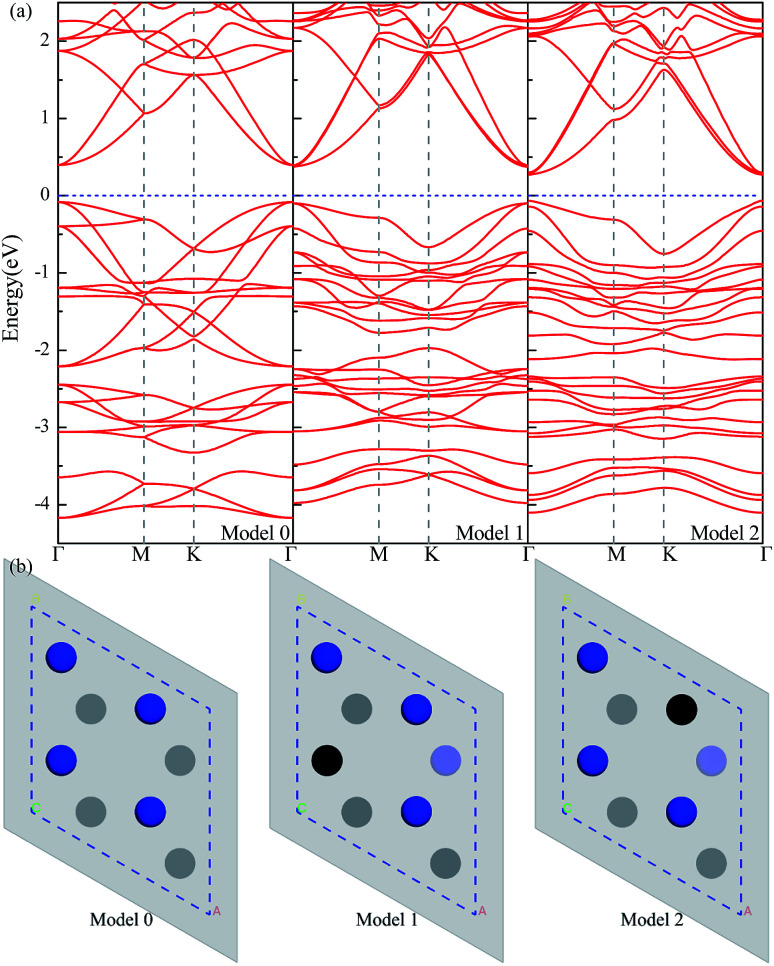
(a) Band structures of Sc_3_(CN)F_2_ in Model 0, 1 and 2, the Fermi level located at zero; (b) the C (black) and N (blue) atoms arrangement models sketch, atoms under the gray semi-transparent interfaces present the second layer atoms.

With the semiconducting MXene Sc_3_(CN)F_2_ investigated in this work, its carrier mobilities with all the required parameters are then calculated and given in Table 2. From the table, the electron mobility of Sc_3_(CN)F_2_ by CBM, the red curve in Fig. 1(d), appears to be highly anisotropic, *i.e.* 1.348 × 10^3^ cm^2^ V^−1^ s^−1^ along the *x* (*Γ* → *X*) and 0.319 × 10^3^ cm^2^ V^−1^ s^−1^ along the *y* (*Γ* → *Y*) directions, respectively. For the hole mobilities, two quasi-degenerated sub-bands are present at the VBM as Fig. 1(d) indicates, and we distinguish the two sub-bands as “h1” (orange) and “h2” (blue), respectively. Both of the sub-bands have been calculated, and the total hole mobilities can be estimated as the statistical average of the two sub-bands on the basis of the Boltzmann distribution. Accordingly, the hole mobilities are determined to be 0.078 × 10^3^ along the *x* and along the *y* directions for the “h1” sub-band, and are 0.956 × 10^3^ along the *x* and 1.003 × 10^3^ cm^2^ V^−1^ s^−1^ along the *y* directions for the “h2” sub-band, respectively. From Table 2, one may note that, for the “h1” or “h2” sub-band, the values of the effective mass and deformation potential constant along the *x* and *y* directions are close to each other, analogous to the Sc_2_CT_2_ MXenes calculated in our previous work.^54^ The average hole mobilities of Sc_3_(CN)F_2_ are 0.517 × 10^3^ along *x* and 0.540 × 10^3^ cm^2^ V^−1^ s^−1^ along the *y* directions, respectively. Consequently, the predicted hole mobilities for Sc_3_(CN)F_2_ are almost isotropic. The details of the carrier effective mass calculations are provided in the ESI.† Actually, the electron mobilities are slightly lower than that of Sc_2_CF_2_ and Sc_2_C(OH)_2_, while the hole mobilities are higher than that of Sc_2_CF_2_ and Sc_2_C(OH)_2_.^30^ The predicted carrier mobilities are much higher than that of monolayer MoS_2_,^58^ providing a hopeful application in nanoelectronics devices for the Sc_3_(CN)F_2_ MXene. Moreover, in order to exclude the impact of structural disorder, *i.e.* the entropy effect on the semiconducting nature of Sc_3_(CN)F_2_, the possibilities of a random distribution of C and N atoms are taken into consideration as well. Three 2 × 2 × 1 super-cells with different C and N arrangement models are built for band structure calculations as shown in Fig. 4. Model 0 represents the ordered arrangement of C and N, and Model 1 and 2 are disordered ones. The results confirm that the Sc_3_(CN)F_2_ MXene is a semiconductor and imply that the random C and N distribution can lead to a slight sub band splitting of CBM and VBM, while the slopes of the bands near the Fermi level keep similar trends, suggesting that the ordered or disordered Sc_3_(CN)F_2_ MXene might have similar carrier mobilities.

**Table 2:** The carrier mobilities of Sc_3_(CN)F_2_. Carrier type “e” and “h” denote “electron” and “hole”, respectively. 
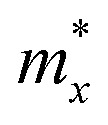
 and 
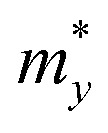
 are the effective masses along the *x* and *y* directions. *E*_*x*_ and *E*_*y*_ are the deformation potential constants, *C*_*x*_ and *C*_*y*_ are the elastic moduli. *μ*_*x*_ and *μ*_*y*_ are the room-temperature carrier mobilities

Carrier type	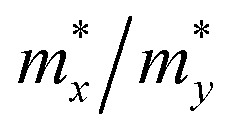	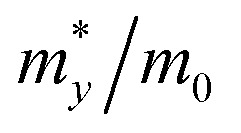	*E*_*x*_ (eV)	*E*_*y*_ (eV)	*C*_*x*_ (J m^−2^)	*C*_*y*_ (J m^−2^)	*μ*_*x*_ (10^3^ cm^2^ V^−1^ s^−1^)	*μ*_*y*_ (10^3^ cm^2^ V^−1^ s^−1^)
e	0.21	1.70	5.722	4.144	262.15	262.17	1.348	0.319
h1	2.93	2.81	4.422	4.622	262.15	262.17	0.078	0.078
h2	0.51	0.53	−3.098	−2.894	262.15	262.17	0.956	1.003

**Fig. 5:**
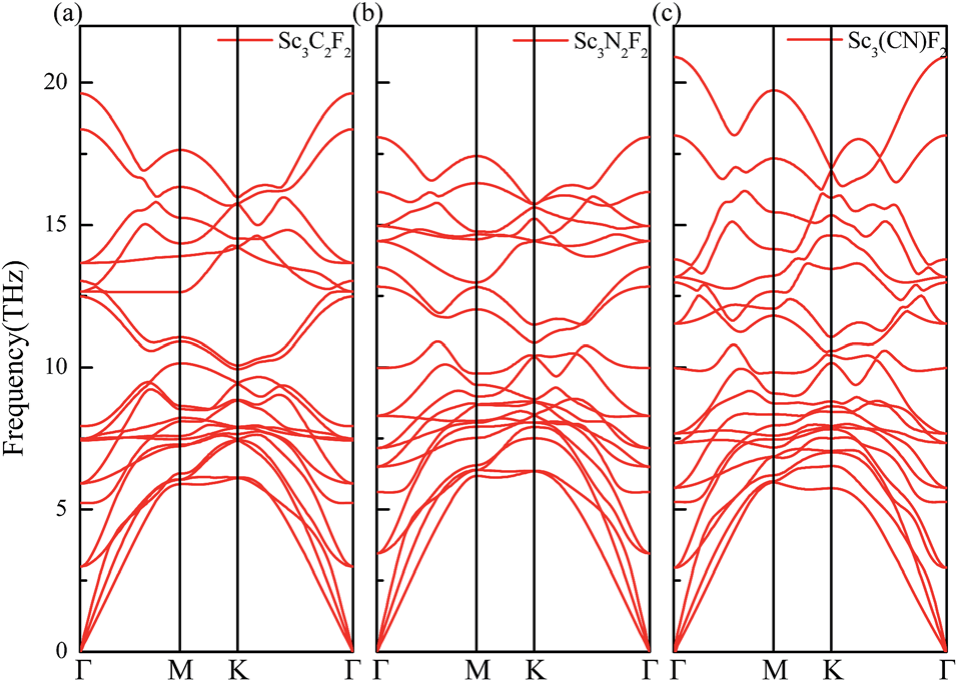
The phonon dispersions of the Sc_3_C_2_F_2_ (a), Sc_3_N_2_F_2_ (b) and Sc_3_(CN)F_2_ (c) MXenes.

The Sc_3_C_2_F_2_, Sc_3_N_2_F_2_ and Sc_3_(CN)F_2_ MXenes phonon dispersions along *Γ* → *M* → *K* → *Γ* are given in Fig. 5. From the figure, the absence of imaginary phonon frequencies implies the structural stabilities of those MXenes. It is well known that thermal conductivities for semiconductive materials are dominantly contributed by phonon transport. Therefore, the lattice thermal conductivities for Sc_3_(CN)F_2_ are thus investigated in the current work and the electronic thermal conductivity for Sc_3_(CN)F_2_ is considered negligible. The values for the Sc_3_(CN)F_2_ MXene are calculated according to eqn (2) based on the phonon dispersions. The required parameters, including the group velocity *υ*_*j*_, Grüneisen parameter *γ*_*j*_ and the square of the Grüneisen parameter 〈*γ*^2^_*j*_〉 are list in Table 3. From the table, the group velocities along the *Γ* → *M* (real-space *y*) direction for transversal acoustic (TA), longitudinal acoustic (LA) and out-of-plane acoustic (ZA) modes are larger than the *Γ* → *K* (real-space *x*) direction. In particular, the group velocity values for the ZA mode along *Γ* → *M* are approximately 20% higher. Moreover, the minimum values for Grüneisen parameter *γ*_*j*_ and 〈*γ*^2^_*j*_〉 found originated from the ZA mode along the *Γ* → *M* direction. For the *Γ* → *K* direction, the minimum in *γ*_*j*_ and 〈*γ*^2^_*j*_〉 occurs in the LA mode. The ratio of 〈*γ*^2^_*j*_〉 between the *Γ* → *K* and *Γ* → *M* directions is the maximum by the ZA mode. These may imply that the out of plane phonon modes are responsible for anisotropy in thermal conductance. Similar phenomena can be found in the parameters for calculating the thermal conductivities of Sc_2_CF_2_, Zr_2_CO_2_ and Hf_2_CO_2_ MXenes. Based on the parameters obtained, the thermal conductivities of Sc_3_(CN)F_2_ have been calculated.

**Table 3:** The group velocity *υ*_*j*_, the Grüneisen parameter *γ*_*j*_ and the square of the Grüneisen parameter 〈*γ*_*j*_^2^〉 for calculating the thermal conductivities of Sc_3_(CN)F_2_

	*υ*_*j*_ (m s^−1^)			*γ*_*j*_			〈*γ*_*j*_^2^〉		
	TA	LA	ZA	TA	LA	ZA	TA	LA	ZA
*Γ* → *M*	3387	3897	3301	2.901	1.189	0.942	8.685	2.254	0.899
*Γ* → *K*	3220	3431	2758	1.909	1.574	1.893	4.263	2.591	4.032

**Fig. 6:**
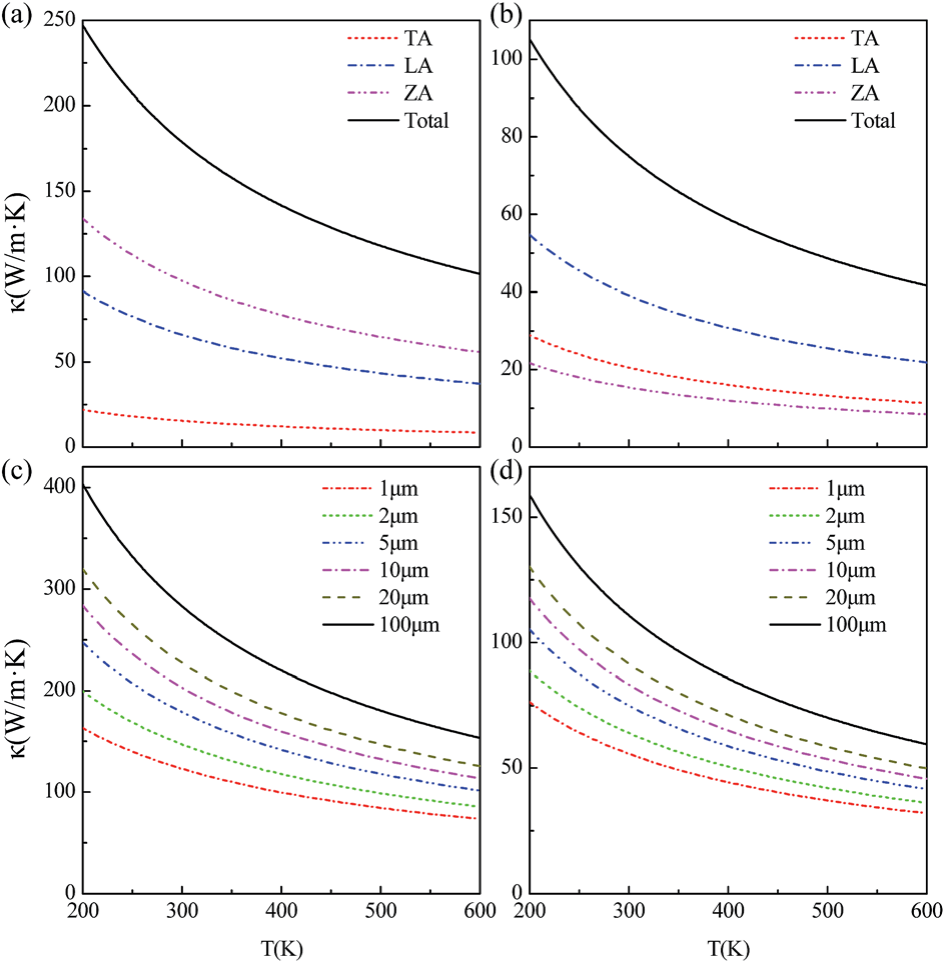
The temperature dependence thermal conductivities for the Sc_3_(CN)F_2_ MXene along the *Γ* → *M* (a) and *Γ* → *K* (b) directions with 5 μm flake length with TA, LA and ZA contributions. The temperature dependence thermal conductivities for the Sc_3_(CN)F_2_ MXene with 1–100 μm flake lengths along the *Γ* → *M* (c) and *Γ* → *K* (d) directions.

The thermal conductivity is dependent upon the flake length *d* due to the existence of boundary scattering. The theoretical temperature dependence thermal conductivity of Sc_3_(CN)F_2_ with flake lengths of 5 μm along the *Γ* → *M* and *Γ* → *K* directions with TA, LA and ZA contributions are plotted in Fig. 6(a) and (b), respectively. The ZA mode has the highest contribution to the theoretical thermal conductivity along the *Γ* → *M* direction, due to the small value for the square of the Grüneisen parameter 〈*γ*^2^_*j*_〉, and the same is for the LA mode along the *Γ* → *M* direction. At room temperature (300 K), the calculated total thermal conductivities with TA, LA and ZA contributions along the *Γ* → *M* and *Γ* → *K* directions are 179 and 75.0 W m^−1^ K^−1^, respectively. The anisotropy in thermal conductivity is similar with that for other MXenes such as Sc_2_CF_2_, Sc_2_C(OH)_2_,^54^ Ti_2_CO_2_, Zr_2_CO_2_ and Hf_2_CO_2_,^55^ demonstrating that anisotropic thermal conductivity may be a common feature for semiconducting MXenes including Sc_3_(CN)F_2_. The temperature dependent thermal conductivities for the Sc_3_(CN)F_2_ MXene with flake lengths of 1–100 μm along the *Γ* → *M* and *Γ* → *K* directions are shown in Fig. 6(c) and (d), respectively. From the figure, the thermal conductivity increases monotonically with increasing flake length in both directions, and is more sensitive to the flake length at low temperatures. The room temperature thermal conductivity along the *Γ* → *M* direction increases from 123 to 283 W m^−1^ K^−1^ as the flake length increases from 1 to 100 μm, which can be understood as analogous to grain size controlled thermal conductivity for bulk materials. Comparatively, the thermal conductivity along the *Γ* → *K* direction increases from 55.7 to 111 W m^−1^ K^−1^, approximately half of that in the *Γ* → *M* direction. Despite that the room temperature thermal conductivity is much lower than the values in the range 4.84 (± 0.44) × 10^3^ to 5.30 (± 0.48) × 10^3^ W m^−1^ K^−1^ for single-layer graphene,^56^ the values for Sc_3_(CN)F_2_ and other MXenes like Sc_2_CT_2_ (T = F, OH) are of the same order of magnitude, higher than that of the phosphorene^59^ and monolayer MoS_2_.^60^ These results indicate that the Sc_3_(CN)F_2_ possesses good heat dissipation performance if used as an electronic device.

**Fig. 7:**
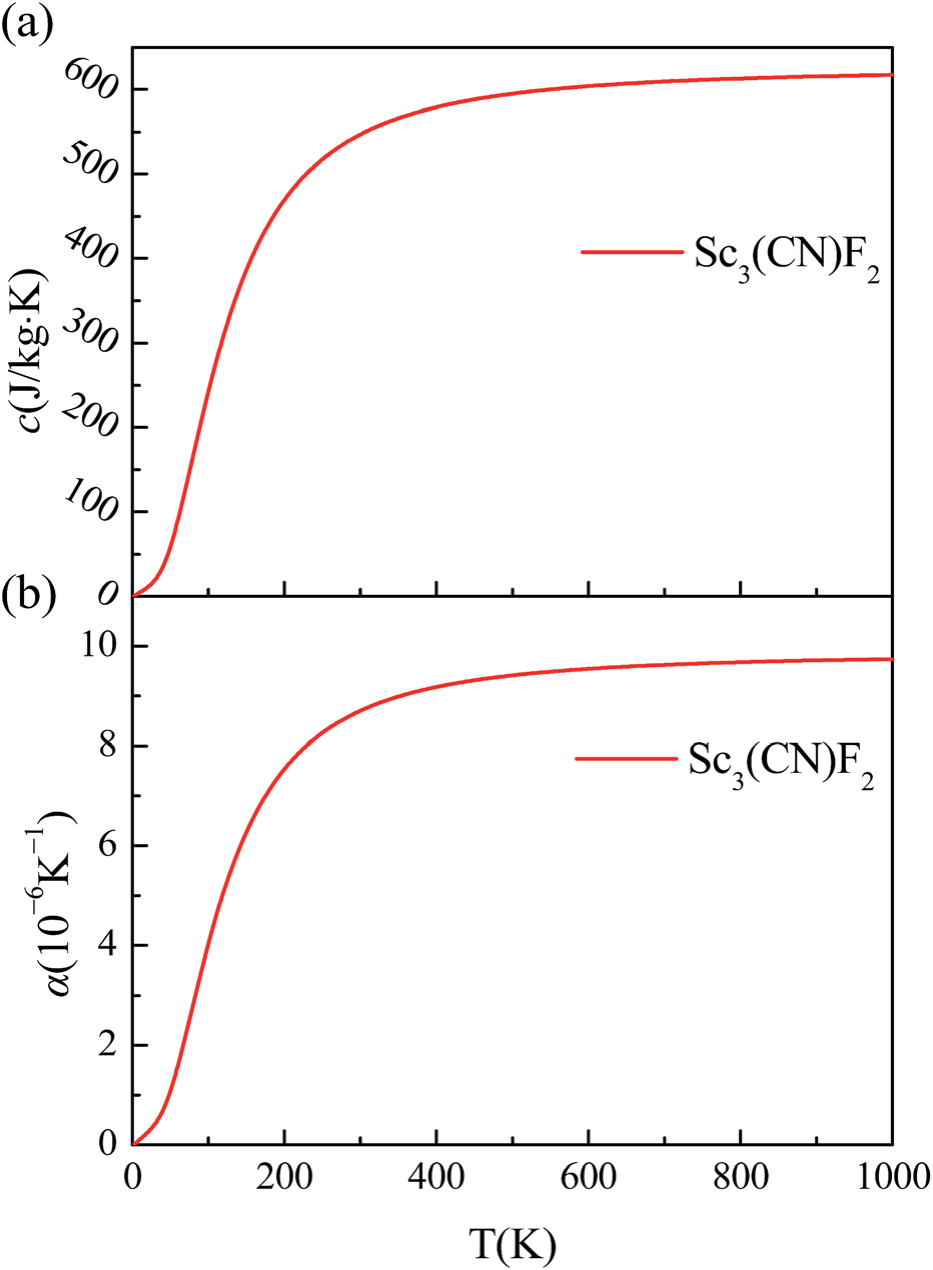
(a) The temperature dependence of Sc_3_(CN)F_2_ specific heat. (b) The temperature dependence of the Sc_3_(CN)F_2_ thermal expansion coefficient.

The specific heat and thermal expansion coefficient are also studied from the phonon dispersion for the hexagonal BZ of Sc_3_(CN)F_2_, and the corresponding temperature dependence for Sc_3_(CN)F_2_ are shown in Fig. 7(a) and (b). These results suggest that both the specific heat and thermal expansion coefficient are positively related to the temperature, and the room temperature values are 547 J kg^−1^ K^−1^ and 8.703 × 10^−6^ K^−1^, respectively. By contrast, the specific heat and thermal expansion coefficient are 385 J kg^−1^ K^−1^ and 16.5 × 10^−6^ K^−1^ for copper, and 412 J kg^−1^ K^−1^ and 11.8 × 10^−6^ K^−1^ for iron. In addition, the room temperature specific heat is much higher than the value of 238 J kg^−1^ K^−1^ due to the relatively small relative atomic mass of Sc and the thermal expansion coefficient is close to the value of 6.094 × 10^−6^ K^−1^ for Hf_2_CO_2_ MXene.^55^ The relatively high specific heat and low thermal expansion coefficient make Sc_3_(CN)F_2_ a good candidate material for nanoelectronic devices.

## Conclusions

In this work, we report our design and theoretical calculations of the semiconducting MXene Sc_3_(CN)F_2_. Different from the mother metallic Sc_3_C_2_F_2_ and Sc_3_N_2_F_2_ MXenes, the Sc_3_(CN)F_2_ MXene is a semiconductor with an indirect band gap of 1.18 eV from the HSE06 band structures analysis. The electrical and thermal properties of the Sc_3_(CN)F_2_ MXene are subsequently predicted by the current computational study. The Sc_3_(CN)F_2_ presents great anisotropy in electron mobility, and approximate isotropy in hole mobility. The electron mobilities of Sc_3_(CN)F_2_ are 1.348 × 10^3^ along *x* and 0.319 × 10^3^ cm^2^ V^−1^ s^−1^ along the *y* directions, and the hole mobilities are 0.517 × 10^3^ along *x* and 0.540 × 10^3^ cm^2^ V^−1^ s^−1^ along the *y* directions, respectively. The thermal conductivities for the Sc_3_(CN)F_2_ are studied with flake lengths of 1–100 μm. The thermal conductivity increases monotonically with increasing flake length, and the room temperature thermal conductivity along the *Γ* → *M* direction is 179 W m^−1^ K^−1^ with a flake length of 5 μm. In addition, the relatively high specific heat and low thermal expansion coefficient make Sc_3_(CN)F_2_ a good candidate material for nanoelectronic devices. The computational data provided here is expected to be meaningful for the expansion of the MXene family towards applications in electronic devices.

## Conflicts of interest

There are no conflicts to declare.

## Supplementary Material

RA-008-C8RA03424A-s001
