# Supplementary material for: First-principles study on the electrical and thermal properties of the semiconducting Sc3(CN)F2 MXene
Source: RSC Adv. 2018 Jun 19;8(40):22452–9. doi: 10.1039/c8ra03424a (PMC9081388; doi:10.1039/c8ra03424a)
Supplement: RA-008-C8RA03424A-s001 [file RA-008-C8RA03424A-s001.pdf]

Electronic Supplementary Material (ESI) for RSC Advances.
This journal is © The Royal Society of Chemistry 2018

First-principles study on the electrical and thermal properties of semiconducting $Sc_3(CN)F_2$ MXene

Kan Luo,[a,b] Xian-Hu Zha,[b] Yuhong Zhou,[b] Zhansheng Guo,[c] Cheng-Te Lin,[d] Qing Huang,[b] Shenghu Zhou,[a,*] Ruifeng Zhang,[e] and Shiyu Du[b,*]

[a]*School of Chemical Engineering, East China University of Science and Technology, Shanghai, China. E-mail address: zhoushenghu@ecust.edu.cn*

[b]*Engineering Laboratory of Specialty Fibers and Nuclear Energy Materials, Ningbo Institute of Materials Technology and Engineering, Chinese Academy of Sciences, Ningbo, Zhejiang, China. E-mail address: dushiyu@nimte.ac.cn*

[c]*Shanghai Institute of Applied Mathematics and Mechanics, Shanghai University, Shanghai, China.*

[d]*Key Laboratory of Marine Materials and Related Technologies, Zhejiang Key Laboratory of Marine Materials and Protective Technologies, Ningbo Institute of Materials Technology and Engineering, Chinese Academy of Sciences, Ningbo, Zhejiang, China.*

[e]*School of Materials Science and Engineering, Beihang University, Beijing, China.*

Table S1 presents the linear fitting data to calculate the deformation potential constant, and the linear fittings for the CBM or VBM $h1$, $h2$ under uniaxial strains are shown in **Fig. S1**. The fourth order polynomial function $y = Intercept + B_1x + B_2x^2 + B_3x^3 + B_4x^4$ is adopted to fit the relationship between the energy data points and the wave vectors along the transport direction for the estimation of the carrier's effective mass. Thirty energy data points near the CBM or VBM (the Γ point $x = 0.1541$ is included) are adopted. With the fitting function, the second derivative value at Γ point is indeed calculated as:

$\frac{\partial 2y}{\partial x2} x = \Gamma = 2B_2 + 6B_3x + 12B_4x^2 | x = \Gamma$. According to the definition of carrier effective mass $m^* = h2\left(\frac{\partial 2y}{\partial x2}\right)-1$, the electron effective mass is calculated. The CBM and VBM energy data points (X→Γ and Γ→Y) polynomial fitting diagrams are shown in **Fig. S2**. The energy data points fitting parameters B_2, B_3 and B_4 for effective masses calculations, the second derivative value at Γ point $\partial^2 y/\partial x^2|_{x=\Gamma}$, the effective mass m^*_e, the electron mass m_0, and the deformation potential constant E are listed in **Table S2**.

Table S1 Linear fitting data to calculate the deformation potential constant.

(eV)		Strain	CBM/VBM−E_F	E_F	CBM/VBM	VACUUM	CBM/VBM−VACUUM
e	*x*	−0.010	0.3054	−1.563	−1.2578	3.1926	−4.4504
		−0.005	0.3281	−1.580	−1.2519	3.1628	−4.4147
		0.000	0.3713	−1.617	−1.2460	3.1451	−4.3911
		0.005	0.3975	−1.638	−1.2400	3.1107	−4.3507
		0.010	0.3935	−1.631	−1.2371	3.1022	−4.3393
	y	−0.010	0.3391	−1.579	−1.2395	3.1846	−4.4241
		−0.005	0.3525	−1.596	−1.2435	3.1565	−4.4000
		0.000	0.3713	−1.617	−1.2460	3.1451	−4.3911
		0.005	0.3885	−1.638	−1.2494	3.1050	−4.3544
		0.010	0.4064	−1.659	−1.2530	3.0903	−4.3433
*h*1	*x*	−0.010	−0.1631	−1.563	−1.7263	3.1926	−4.9189
		−0.005	−0.1466	−1.580	−1.7266	3.1628	−4.8894
		0.000	−0.1098	−1.617	−1.7271	3.1451	−4.8722
		0.005	−0.0902	−1.638	−1.7277	3.1107	−4.8384
		0.010	−0.1010	−1.631	−1.7316	3.1022	−4.8338
	y	−0.010	−0.1480	−1.579	−1.7266	3.1846	−4.9112
		−0.005	−0.1313	−1.596	−1.7273	3.1565	−4.8838
		0.000	−0.1098	−1.617	−1.7271	3.1451	−4.8722
		0.005	−0.0906	−1.638	−1.7285	3.1050	−4.8335
		0.010	−0.0711	−1.659	−1.7305	3.0903	−4.8208
*h*2	*x*	−0.010	−0.0856	−1.563	−1.6488	3.1926	−4.8414
		−0.005	−0.1085	−1.580	−1.6885	3.1628	−4.8513
		0.000	−0.1098	−1.617	−1.7271	3.1451	−4.8722
		0.005	−0.1273	−1.638	−1.7648	3.1107	−4.8755
		0.010	−0.1739	−1.631	−1.8045	3.1022	−4.9067
	y	−0.010	−0.0713	−1.579	−1.6499	3.1846	−4.8345
		−0.005	−0.0933	−1.596	−1.6893	3.1565	−4.8458
		0.000	−0.1098	−1.617	−1.7271	3.1451	−4.8722
		0.005	−0.1278	−1.638	−1.7657	3.1050	−4.8707
		0.010	−0.1447	−1.659	−1.8041	3.0903	−4.8944

Table S2 The specific CBM or VBM energy data points fitting parameters for effective masses calculations. B_2, B_3, and B_4 are fitting parameters, $\partial^2 y/\partial x^2|_{x=\Gamma}$ is the second derivative value at Γ point, m_e^* is the effective mass, m_0 is the electron mass, and E is the deformation potential constant.

Carrier type	Direction	B_2	B_3	B_4	$\partial^2 y/\partial x^2\|_{x=\Gamma}$	m_e^* (×10^31 kg)	m_e^*/m_0	E(eV)
e	*x*	7425	−41245	86700	1425	1.923	0.21	5.722
	y	−1143	5057	−7763	176.8	15.50	1.70	4.144
*h*1	*x*	−195.1	556.8	−797.4	−102.5	26.73	2.93	4.422
	y	−24.26	−290.2	735.7	−107.2	25.57	2.81	4.622
*h*2	*x*	1672	−7397	10193	−591.0	4.637	0.51	−3.098
	y	2527	−12439	20640	−565.4	4.847	0.53	−2.894

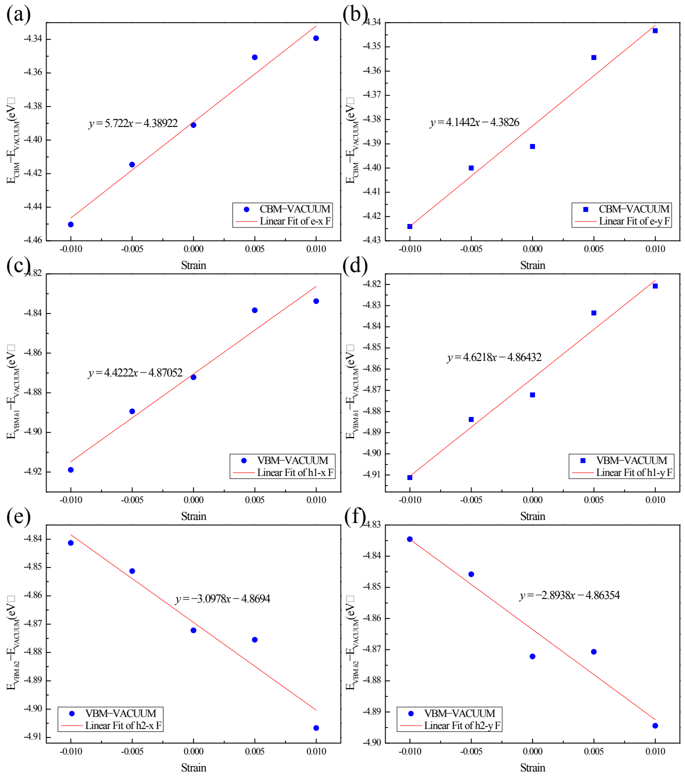

Fig. S1 Linear fitting for the CBM (along the directions of *x* (a) and *y* (b)) or VBM *h*1 (along the directions of *x* (c) and *y* (d)), *h*2 (along the directions of *x* (e) and *y* (f)) under uniaxial strains.

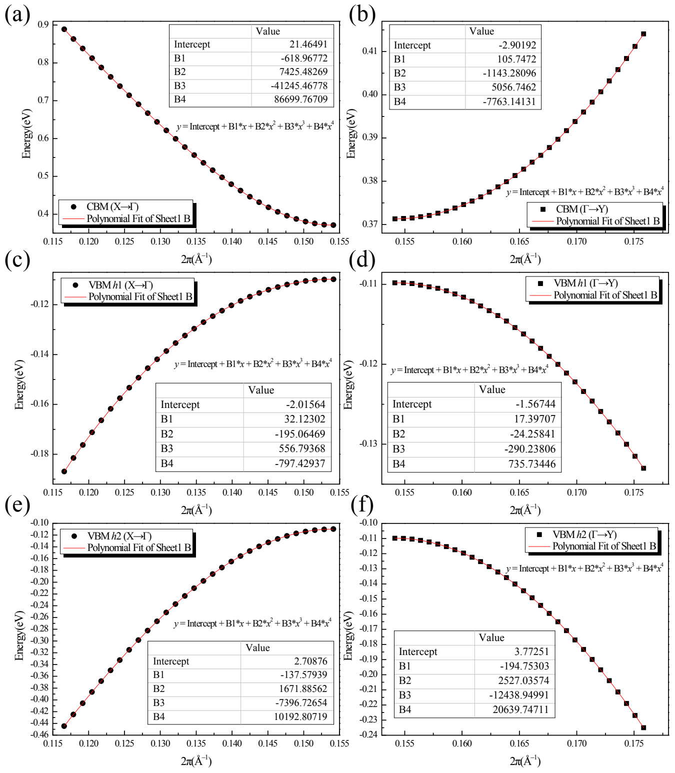

Fig. S2 The polynomial fitting for the CBM (Γ→X (a) and Γ→Y (b)) and VBM *h*1 (Γ→X (c) and Γ→Y (d)), *h*2 (Γ→X (e) and Γ→Y (f)) energy data points.

Optimized $Sc_3C_2F_2$

```
Sc3C2F2 # Please generate POTCAR
   1.00000000000000
     3.2428701161833362    0.0000000000000000    0.0000000000000000
    -1.6214350580916681    2.8084079017881591    0.0000000000000000
     0.0000000000000000    0.0000000000000000   30.0054383522162595
   Sc   C    F
     3     2     2
Direct
  0.6666666666666643  0.3333333333333357  0.5898118707489317
  0.3333333333333357  0.6666666666666643  0.4101881292510754
  0.0000000000000000  0.0000000000000000  0.5000000000000000
  0.6666666666666643  0.3333333333333357  0.4529075454323319
  0.3333333333333357  0.6666666666666643  0.5470924545676752
  0.0000000000000000  0.0000000000000000  0.3722785198271623
  0.0000000000000000  0.0000000000000000  0.6277214801728448
```

Optimized $Sc_3(CN)F_2$

```
Sc3CNF2 # Please generate POTCAR
   1.00000000000000
     3.2436710298252809    0.0000000000000000    0.0000000000000000
    -1.6218355149126404    2.8091015133483213    0.0000000000000000
     0.0000000000000000    0.0000000000000000   30.0038464469823509
   Sc   C    N    F
     3     1     1     2
Direct
  0.6666666666666643  0.3333333333333357  0.5844641503799650
  0.3333333333333357  0.6666666666666643  0.4136135323868189
  0.0000000000000000  0.0000000000000000  0.4981411371576812
  0.6666666666666643  0.3333333333333357  0.4560292851414331
  0.3333333333333357  0.6666666666666643  0.5489362737651788
  0.0000000000000000  0.0000000000000000  0.3758545802140176
  0.0000000000000000  0.0000000000000000  0.6229610409549480
```

Optimized $Sc_3N_2F_2$

```
Sc3N2F2 # Please generate POTCAR
   1.00000000000000
     3.1895811664885794    0.0000000000000000    0.0000000000000000
```

-1.5947905832442897 2.7622583176115088 0.0000000000000000
0.0000000000000000 0.0000000000000000 30.0002247438099623
Sc N F
3 2 2
Direct
0.6666666666666643 0.3333333333333357 0.5832997146798533
0.3333333333333357 0.6666666666666643 0.4167002853201538
0.0000000000000000 0.0000000000000000 0.5000000000000000
0.6666666666666643 0.3333333333333357 0.4548675197202030
0.3333333333333357 0.6666666666666643 0.5451324802798041
0.0000000000000000 0.0000000000000000 0.3775225975908683
0.0000000000000000 0.0000000000000000 0.6224774024091388

Optimized $Sc_3(CN)F_2$ based on an orthorhombic unit cell
Sc3CNF2
1.00000000000000
3.2436712763298088 0.0000000000000000 0.0000000000000000
0.0000000000000000 5.6182034536550161 0.0000000000000000
0.0000000000000000 0.0000000000000000 30.0000000000000000
Sc C N F
6 2 2 4
Direct
0.5000000000000000 0.1666685532117782 0.5844726890884644
0.0000000000000000 0.6666685822117770 0.5844726890884644
0.0000000000000000 0.3333358719148336 0.4136012950260337
0.5000000000000000 0.8333358429148348 0.4136012950260337
0.0000000000000000 0.0000185392148424 0.4981425315030776
0.5000000000000000 0.5000185392148424 0.4981425315030776
0.5000000000000000 0.1666610880145640 0.4560221503595585
0.0000000000000000 0.6666611170145629 0.4560221503595585
0.0000000000000000 0.3333282471532968 0.5489388196952802
0.5000000000000000 0.8333282181532979 0.5489388196952802
0.0000000000000000 0.9999907824590508 0.3758382280206973
0.5000000000000000 0.4999907824590508 0.3758382280206973
0.0000000000000000 0.9999969180316342 0.6229743013068969
0.5000000000000000 0.4999969180316342 0.6229743013068969
